# Fractalkine restores the decreased expression of StAR and progesterone in granulosa cells from patients with polycystic ovary syndrome

**DOI:** 10.1038/srep26205

**Published:** 2016-07-08

**Authors:** Shuo Huang, Yanli Pang, Jie Yan, Shengli Lin, Yue Zhao, Li Lei, Liying Yan, Rong Li, Caihong Ma, Jie Qiao

**Affiliations:** 1 Center for Reproductive Medicine, Department of Obstetrics and Gynecology, Peking University Third Hospital, Beijing, China; 2 Key Laboratory of Assisted Reproduction, Ministry of Education, Beijing, China; 3 Beijing Key Laboratory of Reproductive Endocrinology and Assisted Reproductive Technology, Beijing, China

## Abstract

Low progesterone levels are associated with luteal phase deficiency in women with polycystic ovary syndrome (PCOS). The mechanisms regulating progesterone biosynthesis in the granulosa cells from women with PCOS is largely unknown. Fractalkine is expressed in human ovaries, and is reported to regulate progesterone production in granulosa cells of healthy women. In the current study, we aimed to examine the role of fractalkine in women with PCOS. Reduced fractalkine levels were found in follicular fluid and granulosa cells, accompanied by decreased progesterone production and reduced steroidogenic acute regulatory protein (StAR) expression in the granulosa cells of patients with PCOS. Administration of fractalkine reversed the inhibition of progesterone and StAR expression. The mechanism mediating these effects may be associated with the inhibition of ERK activity in the granulosa cells from women with PCOS. Our findings revealed that fractalkine regulated steroidogenesis in follicular granulosa cells of women with PCOS.

Polycystic ovary syndrome (PCOS) is one of the most common endocrine disorders affecting 5–10% of women of reproductive age^1^,^2^. PCOS is characterized by clinical and/or biochemical hyperandrogenism, oligo/anovulation, and polycystic ovaries. The major complaint of women of reproductive age with PCOS is infertility^3^,^4^.

Progesterone produced by ovarian granulosa cells plays an important role in the maintenance of pregnancy after ovulation and during early pregnancy until placental function is established^5^. Women with PCOS have low levels of progesterone and high spontaneous abortion rates^6^,^7^,^8^. These characteristics may be related to oligo/anovulation-induced corpus luteum dysfunction. However, to the best of our knowledge, the detail mechanisms mediating the reduced levels of progesterone in women with PCOS are poorly understood.

Fractalkine (CX3CL1) and its receptor CX3CR1 are involved in cell adhesion and signal transduction^9^,^10^. Fractalkine is expressed in the ovary and augments progesterone biosynthesis by luteinizing granulosa cells in rats^11^. Our previous data showed the expression of fractalkine and CX3CR1 in human ovaries and demonstrated that fractalkine enhances progesterone biosynthesis in human luteinized granulosa cells, probably by increasing the expression of steroidogenesis-associated proteins^12^. However, no studies have eximined the expression and function of fractalkine in women with PCOS.

Therefore, in this study, we aimed to determine the expression level of fractalkine in women with PCOS and to explore whether administration of fractalkine to isolated luteinized granulosa cells would rescue the impaired biosynthesis of progesterone in women with PCOS. Our data provided important insights into the mechanisms through which fractalkine regulates progesterone expression in granulosa cells.

## Results

### Clinical Characteristics of the patients

Women with PCOS and controls have no significant differences in age or BMI (Table 1). They were all on their first *in vitro* fertilization (IVF) cycle and were received a standard gonadotropin releasing hormone (GnRH) antagonist protocol when involved in this study. There were no differences on the duration and total dose of gonadotropin (Gn) application, or the levels of estradiol and progesterone on human chorionic gonadotropin (hCG) day between control individuals and patients with PCOS (Table 1).

### Reduced fractalkine levels in follicular fluid and granulosa cells of patients with PCOS

**Figure 1:**
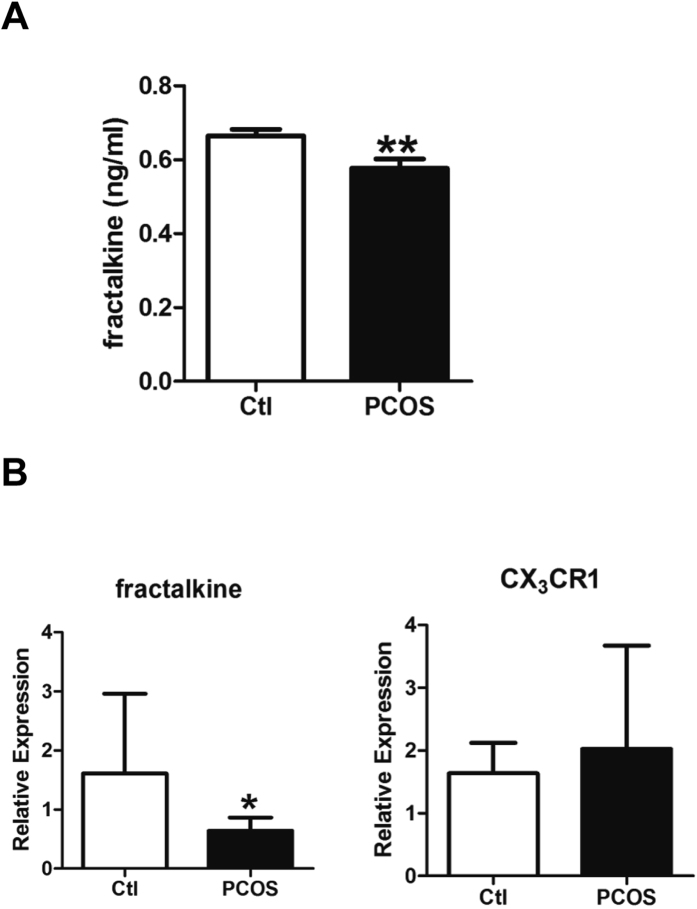
Fractalkine expression was reduced in patients with PCOS. (**A**) The first tube of clear follicular fluid was obtained from patients undergoing IVF-ET, and the levels of fractalkine were measured by ELISA (n = 21 and 16 for Ctl and PCOS, respectively). (**B**) Mural granulosa cells were extracted from follicular fluid by density gradient centrifugation. The levels of transcripts encoding fractalkine and *CX3CR1* were detected by real-time PCR. (n = 6 and n = 10 for Ctl and PCOS, respectively). *P < 0.05, **P < 0.01.

To evaluate the expression levels of fractalkine in patients with PCOS, we detected the secreted fractalkine levels in the follicular fluid. Women with PCOS had significantly reduced fractalkine secretion in the follicular fluid than control (Ctl) individuals (0.577 ± 0.025 versus 0.664 ± 0.018 ng/mL, respectively; *P* < 0.01, n = 16 and n = 21, respectively; Fig. 1A). Mural granulosa cells were extracted from follicular fluid of Ctl individuals and patients with PCOS. The expression levels of fractalkine and *CX3CR1* mRNAs were detected by real-time PCR. Granulosa cells from women with PCOS had significantly reduced expression of fractalkine compared with those from Ctl individuals (0.642 ± 0.070 versus 1.610 ± 0.551, respectively; *P* < 0.05; n = 10 and n = 6, respectively; Fig. 1B). However, there were no changes in *CX3CR1* expression (2.027 ± 0.520 versus 1.637 ± 0.198, respectively; *P* > 0.05; n = 10 and n = 6, respectively; Fig. 1B). These data demonstrated that in women with PCOS, the local expression of fractalkine was downregulated in ovary follicular fluid and mural granulosa cells.

### Decreased progesterone secretion and StAR expression in granulosa cells from patients with PCOS

**Figure 2:**
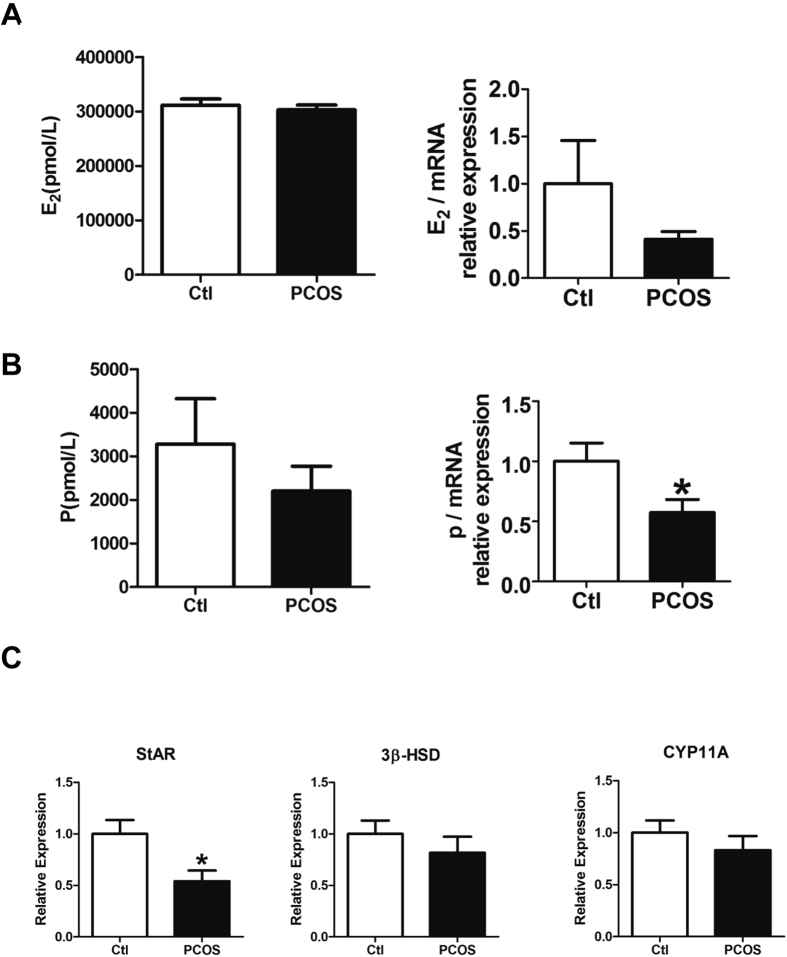
Decreased progesterone secretion and *STAR* expression in granulosa cells from patients with PCOS. (**A**,**B**) Granulosa cells were cultured with 20 ng/mL hCG and 10^−7^ M androstenedione for 48 h. Estradiol (E_2_) and progesterone (P) levels in the culture medium were measured. Estradiol and progesterone levels were normalized according to the level of mRNA. (n = 8 pairs). (**C**) The levels of transcripts encoding StAR, 3β-HSD, and CYP11A in the attached granulosa cells were detected by real-time PCR (n = 14 and n = 17 for Ctl and PCOS, respectively). *P < 0.05.

Next, we administered 20 ng/mL hCG to prime the hormone expression in isolated granulosa cells with 10^−7^ M androstenedione as the substrate for 48 h as performed previously^12^. Estradiol and progesterone levels in the culture medium were measured. The levels of estradiol and progesterone levels were normalized by mRNA to eliminate the interference of cell number variation. Women with PCOS exhibited slightly reduced estradiol levels (0.414 ± 0.079 versus 1.000 ± 0.458, respectively; *P* > 0.05; n = 8; Fig. 2A) and significantly decreased progesterone levels (0.575 ± 0.106 versus 1.000 ± 0.151, respectively; *P* < 0.05; n = 8; Fig. 2B) compared with that in healthy Ctl individuals. To investigate the upstream factors regulating the production of progesterone, the levels of transcripts encoding three key steroidogenesis-associated proteins, i.e., StAR, 3β-HSD, and CYP11A, were measured in attached granulosa cells. Among the three proteins, *STAR* levels were significantly reduced in the PCOS group compared with those in the Ctl group, while no changes in *HSD3B* or *CYP11A1* levels were observed (*STAR*: 0.542 ± 0.103 versus 1.000 ± 0.135, respectively; *P* < 0.05; *HSD3B*: 0.818 ± 0.154 versus 1.000 ± 0.130, respectively; *P* > 0.05; *CYP11A1*: 0.833 ± 0.136 versus 1.000 ± 0.117, respectively; *P* > 0.05; n = 17 and n = 14, respectively; Fig. 2C). Taken together, these data showed that progesterone were down-regulated, probably through reducing expression of the upstream steroidogenesis-associated protein StAR in patients with PCOS.

### Fractalkine treatment increased progesterone and StAR levels in granulosa cells *in vitro*

**Figure 3:**
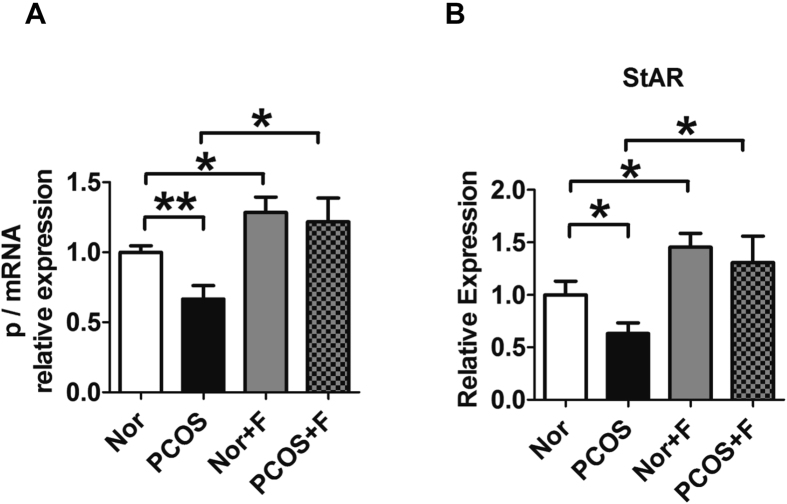
Increased progesterone and *STAR* levels in granulosa cells following treatment with fractalkine *in vitro*. Granulosa cells from women with PCOS or control women were culture with or without 100 ng/mL fractalkine for 48 h. Progesterone levels in the medium (**A**) and *STAR* levels in granulosa cells were measured (**B**) (n = 8 for progesterone expression, n = 7 for *STAR* expression). *P < 0.05, **P < 0.01.

Previously, we reported that fractalkine regulates the expression of progesterone and steroidogenesis-associated proteins. To elucidate the role of fractalkine in mediating progesterone and *STAR* expression in women with PCOS, isolated granulosa cells were cultured with 100 ng/mL fractalkine for 48 h. The dose of fractalkine was selected for the best induction of progesterone based on our previous data^12^. Progesterone levels in the medium and *STAR* levels in granulosa cells were then measured. Fractalkine administration upregulated the expression of both progesterone and *STAR* in Ctl individuals and patients with PCOS. As shown in Fig. 2, compared with Ctl individuals, patients in the PCOS group exhibited decreased expression of progesterone (0.667 ± 0.096 versus 1.000 ± 0.047 for PCOS versus Ctl groups, respectively; *P* < 0.01; n = 8; Fig. 3A) and *STAR* (0.631 ± 0.100 versus 1.000 ± 0.130 for PCOS versus Ctl, respectively; *P* < 0.05; n = 7; Fig. 3B). However, administration of fractalkine blocked this reduction in progesterone (1.218 ± 0.169 versus 1.284 ± 0.110 for PCOS with fractalkine versus Ctl with fractalkine, respectively *P* > 0.05; n = 8; Fig. 3A) and *STAR* expressions (1.306 ± 0.253 versus 1.454 ± 0.131 for PCOS with fractalkine versus Ctl with fractalkine, respectively; *P* > 0.05; n = 7; Fig. 3B) in PCOS groups. These data indicated that administration of fractalkine reversed low levels of progesterone in the granulosa cells from PCOS women. This might be mediated by enhanced expression of StAR.

### Reduced phosphorylation of ERK in PCOS granulosa cells following treatment with fractalkine *in vitro*

**Figure 4:**
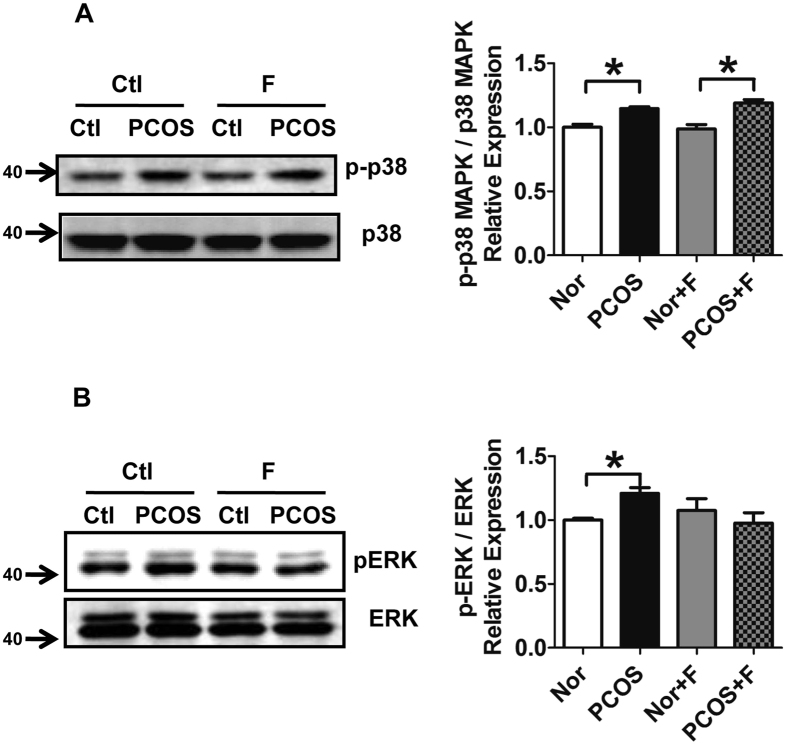
Reduced phosphorylation of ERK in PCOS granulosa cells following treatment with fractalkine *in vitro*. Phosphorylated p38, p38 (**A**), phosphorylated ERK, and ERK (**B**) were detected by western blotting in granulosa cells from women with PCOS and control women with or without fractalkine (100 ng/mL) treatment for 2 h. Quantitative results from two independent experiments are shown on the right. Each sample was a pool from 3–4 patients. *P < 0.05.

The mitogen-activated protein kinase (MAPK) signaling pathways are associated with the regulation of steroidogenesis^13^. To evaluate whether fractalkine regulated MAPK signaling pathways, the MAPK family member p38 MAPK and ERK1/2 were detected in granulosa cells of women with PCOS. The phosphorylation of p38 was significantly enhanced in granulosa cells from patients with PCOS compared with those in healthy individuals (1.146 ± 0.015 versus 1.000 ± 0.024, respectively; *P* < 0.05; Fig. 4A). Administration of fractalkine did not interfere with the expression pattern of phosphorylated p38 (1.190 ± 0.026 versus 0.986 ± 0.035 for PCOS with fractalkine versus Ctl with fractalkine, respectively, *P* < 0.05; 0.986 ± 0.035 versus 1.000 ± 0.024 for Ctl with fractalkine versus Ctl, respectively, *P* = 0.778; 1.190 ± 0.026 versus 1.146 ± 0.015 for PCOS with fractalkine versus PCOS, respectively, *P* = 0.274; Fig. 4A). Granulosa cells from patients with PCOS exhibited increased phosphorylation of ERK compared with those of healthy individuals (1.209 ± 0.045 versus 1.000 ± 0.015, respectively; *P* < 0.05; Fig. 4B). Administration of fractalkine blocked the phosphorylation of ERK in granulosa cells from women with PCOS compared with that in healthy individuals (0.977 ± 0.081 versus 1.076 ± 0.091 for PCOS with fractalkine versus Ctl with fractalkine, respectively; *P* > 0.05; Fig. 4B). These data demonstrated that the phosphorylation of p38 and ERK was increased in granulosa cells from patients with PCOS and that fractalkine repressed the phosphorylation of ERK. It is known that ERK1/2 represses the expression of STAR. Our results showed that fractalkine inhibited the activity of ERK1/2. This might cause the enhanced the expression of STAR, which promoted the production of progesterone in human granulosa cells.

## Discussion

**Figure 5:**
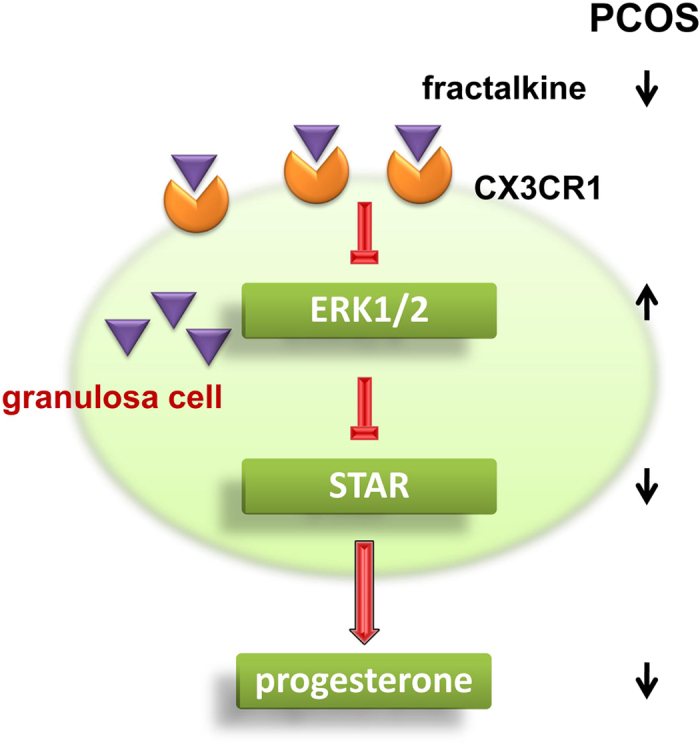
Schematic hypotheses for the mechanisms underlying fractalkine regulated progesterone production in human granulosa cells. It is known that ERK1/2 represses the expression of STAR. Our results show that fractalkine inhibits the activity of ERK1/2, subsequently enhances the expression of STAR, which is known to promote the production of progesterone in human granulosa cells. Through this mechanism, low levels of fractalkine drive the low production of progesterone in the granulosa cells of women with PCOS.

In this study, we examined the role of fractalkine in mediating hormone levels in women with PCOS. It is known that ERK1/2 represses the expression of STAR. Our results showed that fractalkine inhibited the activity of ERK1/2, subsequently enhanced the expression of STAR, which is known to promote the production of progesterone in human granulosa cells. Through this mechanism, low levels of fractalkine drived the low production of progesterone in the granulosa cells of women with PCOS (Fig. 5).

As a member of the CX3C chemokine subclass, fractalkine is a transmembrane molecule composed of 373 amino acids^9^. The major function of fractalkine is its chemoattractive activity for monocytes, natural killer (NK) cells, and T cells^14^. Fractalkine and its receptor CX3CR1 are actively involved in many diseases such as atherosclerosis, rheumatoid arthritis, HIV infection, and cancer^14^,^15^,^16^,^17^. Few reports have described the role of fractalkine in reproduction-related physiological or pathological processes. Fractalkine and CX3CR1 are expressed on the glandular epithelium, decidualized stroma, macrophages, and NK cells or neutrophils in the human endometrium, where they may mediate leukocyte recruitment^18^. In a recent study, fractalkine secretion was shown to be similar in endometrial stromal fibroblasts of women with PCOS and healthy women^19^. Therefore, it is likely that our findings showing reduced levels of fractalkine in granulosa cells and follicular fluid were specific to the ovaries in women with PCOS.

Several studies have shown that women with PCOS have reduced levels of progesterone^7^,^20^,^21^. Progesterone deficiency is associated with habitual abortion^22^. Moreover, women with PCOS often exhibit luteal phase deficiency and increased spontaneous abortions^6^. Therefore, improving our understanding of the factors affecting progesterone production is important for developing improved therapeutic strategies for low levels of progesterone in patients with PCOS. Progesterone is generated from cholesterol, and its biosynthesis is controlled by several proteins, including StAR, 3β-HSD, and CYP11A^23^. In the current study, we found that *STAR* was downregulated in granulosa cells from women with PCOS. The reduced expression of *STAR* may be associated with the impaired progesterone production.

Fractalkine has been shown to be expressed in the ovaries and to enhance the production of progesterone in luteinizing granulosa cells in rats and humans^11^,^12^. Our study revealed the expression and potential role of fractalkine in women with PCOS. Administration of fractalkine reversed the downregulation of progesterone and *STAR* in granulosa cells from patients with PCOS. This result indicated that women with PCOS may benefit from local exposure to fractalkine.

MAPKs signaling pathways have been reported to be associated with the regulation of StAR in steroidogenic tissues^13^. One MAPK, p38 was found to be activated by fractalkine in rat granulosa cells^11^. Moreover, our data showed that fractalkine administration did not interfere with p38 activation. However, ERK, another MAPK, was inhibited by fractalkine. Our findings were consistent with reports showing that the ERK1/2 pathway is activated in ovarian cysts in PKBβ-knockout mice, a PCOS-like mouse model^24^ and that the ERK pathway inhibits StAR expression and consequent progesterone synthesis in rat granulosa cell-derived cell lines and in luteinized human granulosa cells^25^,^26^. The ERK pathway is a negative regulator of progesterone production regulated by prostaglandin F 2α (PGF_2α_) or gonadotropin-releasing hormone agonist (GnRHa) in human granulosa cells. In these studies, administration of ERK inhibitors alone did not interfere with progesterone production in human granulosa cells; however, ERK inhibitors rescued the reduction in progesterone induced by PGF or GnRHa^27^,^28^. Our data indicated that fractalkine inhibited the ERK pathway, which is known to repress StAR expression and progesterone synthesis. However, further studies are needed to clarify the role of ERK in fractalkine-regulated steroidogenesis in women with PCOS.

In summary, the results of the current study provide insights into the regulation of steroidogenesis in follicular granulosa cells from women with PCOS and suggest a novel role for fractalkine in the pathophysiology of PCOS.

## Methods

### Patients

The experimental protocol was approved by the ethics committee of Peking University Health Science Center. The methods were carried out in accordance with the approved guidelines. Written informed consent was obtained from all subjects. Human luteinized granulosa cells were isolated from follicular aspirates of 41 control individuals and 38 women with PCOS (ages 25–38 years) undergoing *in vitro* fertilization and embryo transfer (IVF-ET) at the Reproductive Center of Peking University Third Hospital. The control women had regular menstrual cycles and underwent IVF-ET because of infertility arising from their male partners. The diagnosis of PCOS was based on the Rotterdam-PCOS criteria in which at least two of the following features were present: oligo/amenorrhea, clinical or biochemical hyperandrogenism, and polycystic ovaries on ultrasonography. Women with a diagnosis of endometriosis were excluded. All the PCOS and control patients were on their first IVF cycle and were received a standard GnRH antagonist protocol as described previously^29^. Briefly, on day 2 of a menstrual cycle, gonadotropin (recombinant human follicle-stimulating hormone) was administrated every day with a start dose of 150–225 IU. Starting on day 8, 0.25 mg of GnRH antagonist (Cetrorelix Acetate, Baxter Oncology GmbH, Germany) per day was added into the protocol for injection. When at least three follicles reached 18 mm in diameter, 250 ug Recombinant Human Chorionic Gonadotropin Alfa (Ovidrel, Merck Serono Co., Germany) was administered through subcutaneously injection. Oocyte retrieval was performed 36 h later under transvaginal ultrasound guidance. Follicular fluid used for the measurement of fractalkine level was obtained from the first follicle aspirated from each ovary. After oocyte retrieval, the cells and follicular fluid left in the plates were collected for granulosa cell isolation.

### Reagents

Fractalkine was purchased from R&D Systems Inc. (Minneapolis, MN, USA). Antibodies against phosphorylated p38, p38, phosphorylated extracellular signal-regulated kinase (ERK), and ERK were from Cell Signaling Technology (Danvers, MA, USA).

### Isolation and culture of granulosa cells

Follicular fluid was aspirated from women undergoing IVF-ET. The first tube of clear follicular fluid was collected for fractalkine ELISA assays. The other follicular fluid was washed in phosphate-buffered saline (PBS), and centrifuged over Ficoll (Sigma-Aldrich Inc., St. Louis, MO, USA) to remove red blood cells. Granulosa cells were washed again with PBS, and then the cell deposits were then collected and resuspended in McCoy’s 5a medium (Invitrogen, Carlsbad, CA, USA) supplemented with 100 U/mL penicillin, 100 μg/mL streptomycin, and 10^−7^ M androstenedione. The cells were incubated in the plates for 24 h to avoid the interference from human chorionic gonadotropin (hCG) treatment by the IVF-ET protocol. Cells were treated with 20 ng/mL hCG, and recombinant human fractalkine (100 ng/mL) for steroid measurement and reverse transcription polymerase chain reaction (RT-PCR). The cells were cultured in plates treated with 2.5 μg/mL fibronectin (Sigma-Aldrich Inc.) for western blotting of 2-h time course experiments. mRNA and proteins were extracted from attached cells for real-time PCR and western blotting, respectively.

### Steroid measurement

Media were collected 48 h after culture and stored at −80 °C until estradiol (E2) and progesterone (P) determination using chemiluminescence with an Immunoassay System (Siemens Healthcare Diagnostics Inc., Deerfield, IL, USA). The relative expression of the hormones was normalized by the mRNA level of granulosa cells, and the average of the controls was set as 1. The level of mRNA was measure by NANODROP 2000 (Thermo Scientific, Waltham, MD, USA).

### Enzyme-linked immunosorbent assay (ELISA)

Fractalkine levels in follicular fluid were detected using an ELISA kit (R&D systems Inc.).

### Real-time RT-PCR analysis

mRNAs were extracted with Trizol, followed by chloroform and isopropanol. cDNAs were synthesized using a RevertAid first-strand cDNA synthesis kit (Thermo Scientific, Waltham, MD, USA). SYBR Green-based quantitative RT-PCR was carried out to measure the levels of mRNAs encoding fractalkine, and CX3CR1 in isolated granulosa cells and steroidogenic acute regulatory protein (StAR), 3β-hydroxysteroid dehydrogenase (3β-HSD), and cytochrome P45011A (CYP11A) in cultured granulosa cells. Results were analyzed using ABI Prism 7000 SDS software (Applied Biosystems Inc., Foster City, CA, USA). The relative expression of the genes was normalized by the level of housekeeping gene β-Actin, and the average of the controls was set as 1. The following primers were used: fractalkine, sense, 5′-CACCTTCTGCCATCTGACTGT-3′ and antisense, 5′-ATGCCTGGTTCTGTTGATAGTG-3′; CX3CR1, sense, 5′-CTTTGGGACTGTGTTCCTGTC-3′ and antisense, 5′-ACTCTTGGGCTTCTTGCTGTT-3′; StAR, sense, 5′-GAGCAGAAGGGTGTCATCAGG-3′ and antisense, 5′-GCAGGTGGTTGGCAAAATC-3′; 3β-HSD, sense, 5′-CCAGCATCTTCTGTTTCCTGG-3′ and antisense, 5′-AGCTTGGTCTTGTTCTGGAGTTTAG-3′; CYP11A, sense, 5′-TGGAGTCGGTTTATGTCATCG-3′ and antisense, 5′-GGCCACCCGGTCTTTCTT-3′; and β-actin, sense, 5′-TGCCCATCTACGAGGGGTAT-3′ and antisense, 5′- CTTAATGTCACGCACGATTTCC-3′.

### Western blot analysis

Cell extracts containing equal amounts of total protein were resolved by 10% sodium dodecyl sulfate polyacrylamide gel electrophoresis (SDS-PAGE) for western blot analysis. The blots were incubated with primary antibody (1:1000 dilution, overnight incubation) and IRDye 700DX-conjugated secondary antibody (Rockland Inc). Immunofluorescence signals were detected by Odyssey infrared imaging (LI-COR Biosciences, Lincoln, NE, USA). The densities of the bands were measured by Image J software.

### Statistical analysis

Data are expressed as the mean ± SEM. Statistical analysis involved one-way ANOVA for multiple comparisons, then Tukey-Kramer post-hoc testing, and the Student’s unpaired t-test for comparisons between two groups. Data were analyzed by the use of GraphPad Prism software. Differences with *P* values of less than 0.05 were considered statistically significant.

## Additional Information

**How to cite this article**: Huang, S. *et al*. Fractalkine restores the decreased expression of StAR and progesterone in granulosa cells from patients with polycystic ovary syndrome. *Sci. Rep*. **6**, 26205; doi: 10.1038/srep26205 (2016).

## Figures and Tables

**Table 1:** Characteristics and clinical medication of the patients.

	Normal controls(n = 41)	PCOS cases(n = 38)	*P*-value
	Mean ± SD	Mean ± SD	
Age	30.6 ± 3.8	30.3 ± 4.0	0.734^a^
BMI (kg/m^2^)	22.8 ± 3.4	23.4 ± 3.7	0.456^a^
Days of Gn application	11.7 ± 2.0	11.8 ± 3.0	0.805^b^
Total dose of Gn (IU)	2563.1 ± 946.9	2338.2 ± 824.1	0.263^a^
E_2_ level on hCG day (pmol/l)	9488.2 ± 4100.7	9873.2 ± 5977.4	0.941^b^
P level on hCG day (nmol/l)	2.8 ± 1.3	2.4 ± 1.4	0.192^a^

^a^Student’s *t*-test.

^b^Mann-Whitney test (Variance is not equal, nonparametric test).

Gn: gonadotropin; E2: estradiol; P: progesterone; hCG: human chorionic gonadotropin.
